# Recapitulation of pro-inflammatory signature of monocytes with ACVR1A mutation using FOP patient-derived iPSCs

**DOI:** 10.1186/s13023-022-02506-3

**Published:** 2022-09-21

**Authors:** Hirotsugu Maekawa, Yonghui Jin, Megumi Nishio, Shunsuke Kawai, Sanae Nagata, Takeshi Kamakura, Hiroyuki Yoshitomi, Akira Niwa, Megumu K. Saito, Shuichi Matsuda, Junya Toguchida

**Affiliations:** 1 Department of Cell Growth and Differentiation, Center for iPS Cell Research and Application, Kyoto University, Kyoto, Japan; 2 Department of Orthopaedic Surgery, Graduate School of Medicine, Kyoto University, Kyoto, Japan; 3 Department of Regeneration Sciences and Engineering, Institute for Life and Medical Sciences, Kyoto University, Kyoto, Japan; 4 Department of Immunology, Graduate School of Medicine, Kyoto University, Kyoto, Japan; 5 Department of Clinical Application, Center for iPS Cell Research and Application, Kyoto University, Kyoto, Japan; 6 Department of Fundamental Cell Technology, Center for iPS Cell Research and Application, Kyoto University, Kyoto, Japan

**Keywords:** Fibrodysplasia ossificans progressive (FOP), Monocyte, Inflammation, Induced pluripotent stem cell (iPSC), Activin-A, Bone morphogenic protein (BMP)

## Abstract

**Background:**

Fibrodysplasia ossificans progressiva (FOP) is a rare genetic disease characterized by progressive heterotopic ossification (HO) in soft tissues due to a heterozygous mutation of the ACVR1A gene (FOP-ACVR1A), which erroneously transduces the BMP signal by Activin-A. Although inflammation is known to trigger HO in FOP, the role of FOP-ACVR1A on inflammatory cells remains to be elucidated.

**Results:**

We generated immortalized monocytic cell lines from FOP-iPSCs (FOP-ML) and mutation rescued iPSCs (resFOP-ML). Cell morphology was evaluated during the monocyte induction and after immortalization. Fluorescence-activated cell sorting (FACS) was performed to evaluate the cell surface markers CD14 and CD16 on MLs. MLs were stimulated with lipopolysaccharide or Activin-A and the gene expression was evaluated by quantitative PCR and microarray analysis. Histological analysis was performed for HO tissue obtained from wild type mice and FOP-ACVR1A mice which conditionally express human mutant *ACVR1A* gene by doxycycline administration. Without any stimulation, FOP-ML showed the pro-inflammatory signature of CD16+ monocytes with an upregulation of *INHBA* gene, and treatment of resFOP-ML with Activin-A induced an expression profile mimicking that of FOP-ML at baseline. Treatment of FOP-ML with Activin-A further induced the inflammatory profile with an up-regulation of inflammation-associated genes, of which some, but not all, of which were suppressed by corticosteroid. Experiments using an inhibitor for TGFβ or BMP signal demonstrated that Activin-A-induced genes such as *CD16* and *CCL7*, were regulated by both signals, indicating Activin-A transduced dual signals in FOP-ML. A comparison with resFOP-ML identified several down-regulated genes in FOP-ML including *LYVE-1*, which is known to suppress matrix-formation in vivo. The down-regulation of LYVE-1 in HO tissues was confirmed in FOP model mice, verifying the significance of the in vitro experiments.

**Conclusion:**

These results indicate that FOP-ML faithfully recapitulated the phenotype of primary monocytes of FOP and the combination with resFOP-ML is a useful tool to investigate molecular events at the initial inflammation stage of HO in FOP.

**Supplementary Information:**

The online version contains supplementary material available at 10.1186/s13023-022-02506-3.

## Introduction

Fibrodysplasia ossificans progressiva (FOP) is an extremely rare genetic condition characterized by the systemic and progressive development of mature bone tissues in soft tissues such as skeletal muscles, tendons, and ligaments (heterotopic ossification, HO) [1]. The disease-causing gene is *ACVR1A* gene, which encodes a type I BMP receptor [2], and more than 95% of patients carry an identical mutation, R206H [3]. In most cases, HO is initiated by an episode of painful swelling (flare-up), and histological findings by archival biopsy samples have demonstrated the sequential events of HO in FOP [4]. In the earliest stage, mononuclear cells showing the features of mast cells and macrophages infiltrated at the flare-up sites, which is followed by the proliferation of mesenchymal stromal cells (MSCs), the formation of chondroid tissues, and final bone formation. Repetitious flare-up episodes gradually spread HO in the trunk and extremities to cause a serious inhibition of daily activity [5]. This stepwise exaggeration of the disease suggests that factors transducing the BMP signal via mutant ACVR1A at the flare-up are key to inducing the HO. Our previous study using FOP-patient derived induced pluripotent stem cells (FOP-iPSCs) identified Activin-A as a main flare-up factor, which physiologically transduces the TGFβ signal via a receptor complex with ACVR1B, but erroneously transduces the BMP signal via mutant ACVR1A and initiates the process of HO formation [6, 7]. Identical results were reported by another group using transgenic mice harboring human mutant ACVR1A [8]. This pivotal finding describes the molecular mechanism of FOP and provides new strategies to treat this intractable disease, such as blocking Activin-A with a neutralizing antibody, inhibiting the Activin-A signal by a mutant-specific kinase inhibitor, and inhibiting the downstream signal by mTOR inhibitors [8–10].

Although the molecular events after the binding of Activin-A to mutant ACVR1A on precursor cells have been gradually disclosed, those in the initial inflammation stage are still equivocal. Activin-A is known to be involved in inflammation [11], but its role on monocytes with FOP-ACVR1A is not yet clear. Nearly half of patients experienced the formation of new HO without a clear episode of flare-up [5], suggesting an abnormal response to inflammatory signals in FOP patients. The importance of the initial inflammation step was demonstrated in vivo using an FOP mouse model, in which HO formation was inhibited if mast cells/macrophages were depleted by genetic manipulation or their function of was chemically inhibited [12]. A comprehensive immunophenotype analysis of FOP patient monocytes identified several surface markers including CD16 as up-regulated [13]. The involvement of the p38-MAPK axis, but not the canonical SMAD1/5/9 axis in the BMP signal pathway were observed, suggesting that monocytes were activated by this specific pathway [14, 15]. These data indicated that understanding the effect of mutant ACVR1A in monocytes is important for clarifying the initial event of FOP. The limited growth potential of monocytes, however, makes it difficult to conduct this analysis in detail. In addition, differences between individuals, such as genetic background and previous history of anti-inflammatory therapy, including oral corticosteroid, may compromise the evaluation of the effect of mutant ACVR1A on monocytes.

To overcome these issues, here we established immortalized monocyte cell lines from FOP-iPSCs (FOP-ML) and also from mutation rescued FOP-iPSCs (resFOP-ML), in which the mutant residue was replaced by the wild-type [6]. These cell lines enabled us to perform experiments with fewer limitations and precisely evaluate the effect of mutant ACVR1A on monocytes by comparing two cell lines with identical genomic information except the ACVR1A allele. Taking the advantages of these cell lines, here we found that mutant ACVR1A induced a pro-inflammatory signature in monocytes and possibly contributed to the matrix formation by downregulating an inhibitory factor, LYVE-1 (lymphatic vessel endothelial hyaluronan receptor 1).

## Materials and methods

### Cell culture

The FOP-iPSCs used in this study were established from a FOP patient harboring R206H heterozygous mutation in ACVR1 [16], and mutation-corrected resFOP-iPSCs were generated by BAC-based homologous recombination [6]. iPSCs were maintained in StemFit AK02N (Ajinomot) on iMatrix 511 silk (Nippi)-coated dishes.

Monocytes were induced from FOP- and resFOP-iPSCs by a previously described method with some modification [17] and then immortalized using lentivirus vectors containing *BMI1*, *cMYC*, and *MDM2* genes in the presence of polybrene (Sigma) [18, 19]. Immortalized monocyte cell lines (FOP- and resFOP-ML) were maintained in StemPro-34 (Gibco) supplemented with 2 mM L-glutamine (Gibco), 50 ng/mL recombinant human macrophage colony stimulating factor (M-CSF) (R&D Systems), and 50 ng/mL recombinant human granulocyte macrophage colony stimulating factor (GM-CSF) (R&D Systems) [20]. CD14^+^ FOP- and resFOP-ML were collected by magnetic-activated cell sorting (MACS) using anti-human CD14 MicroBeads (Miltenyi Biotec) every time before use in each experiment, as per the manufacturer’s protocol.

### Fluorescence-activated cell sorting (FACS)

FACS was performed by AriaII (BD) according to the manufacturer’s protocol. The antibodies used in the FACS are listed in Additional file 1: Table S1. In all experiments, FACS histograms of isotype controls were similar to those without antibodies; therefore, histograms without antibodies were used as control populations.

### May-Giemsa staining

FOP- and resFOP-ML were seeded onto MAS-GP type A glass slides (Matsunami) and stained with May-Grunwald and Giemsa staining solution (Merck Millipore) in accordance with the manufacturer’s instructions.

### Immunocytochemical staining

FOP- and resFOP-ML were fixed using 2% paraformaldehyde for 10 min and washed with PBS 3 times. 50–100 μL suspensions containing 50,000–100,000 fixed cells were applied directly to the slide (Matsunami), dried at room temperature, and permeabilized with 100% methanol at 4 °C for 10 min. Samples were blocked with Blocking One or Blocking One-P (Nacalai Tesque) for 60 min and then incubated with anti-CD14, CD16, LYVE-1, or p-Smad5 antibody diluted in Can Get Signal Immunostain Solution B (Toyobo) for 16 to 18 h at 4 °C. Next, the samples were washed 3 times in 0.2% Tween-20 (Sigma-Aldrich) in PBS and incubated with Alexa Fluor 488 conjugated donkey anti-mouse IgG secondary antibody (Abcam) and Alexa Fluor 647 conjugate donkey anti-rabbit IgG secondary antibody (Thermo Fisher Scientific) diluted in Can Get Signal Immunostain Solution B for 1 h at room temperature. DAPI (10 μg/mL) was used to counterstain nuclei.

### RNA isolation and quantitative polymerase chain reaction

Total RNA was extracted using an RNeasy Mini Kit (QIAGEN) with DNase treatment to remove genomic DNA. Total RNA (0.3 μg) was reverse transcribed into cDNA with ReverTra Ace (Toyobo) in a total volume of 20 μL. Quantitative PCR (qPCR) was performed with Thunderbird SYBR qPCR Mix (Toyobo) and analyzed with QuantStudio 12 K Flex Real-Time PCR System (Thermo Fisher Scientific). The primers used are listed in Additional file 1: Table S1. β-Actin was used for normalization as an endogenous control in all data.

### Stimulation and inhibition of signals in FOP- and resFOP-ML

For stimulation experiments, cells were seeded at 100,000 cells per well in a 24-well plate. On the next day, FOP- and resFOP-ML were stimulated with 10 μM lipopolysaccharides (LPS) (Sigma-Aldrich) or 100 ng/mL Activin-A (R&D Systems) with or without 1 μM Dexamethasone (Wako). The cells were collected for RNA extraction or immunostaining 4, 12, or 24 h after the reagent stimulation. For the inhibition experiments, cells were stimulated with Activin-A and simultaneously treated with a TGFβ inhibitor (SB431542) or BMP inhibitor (DMH1) for 24 h. The RNAs were then analyzed as described above.

### Microarray analysis

RNA was extracted from FOP- and resFOP-ML stimulated with 10 ng/mL LPS or 100 ng/mL Activin-A for 12 h and unstimulated as a control (n = 3, biological replicates). After the RNA quality was confirmed by the RNA 6000 Nano Kit (Agilent Technologies), all RNA samples were processed using the Ambion WT Expression Kit (Life Technologies), the GeneChip WT Terminal Labeling and Controls Kit, and the GeneChip Hybridization Wash and Stain Kit (Affymetrix) according to the manufacturers’ protocol. Raw CEL files were imported into GeneSpring GX 14.9 software (Agilent Technologies), and the expressions were calculated using the RMA16 algorithm. Heatmaps, principal component analysis (PCA), and Venn diagrams were generated using GeneSpring GX14.9 software. Upstream analysis was performed using Ingenuity Pathway Analysis (IPA) (QIAGEN). Array data were deposited in the NCBI’s Gene Expression Omnibus (GEO) database (GEO GSE 183,525).

### Induction of HO in FOP mice

The establishment of FOP-ACVR1 conditional transgenic mice (FOP mice) was reported previously, in which the expression of mutant ACVR1 gene is induced by the administration of doxycycline [10]. Female mice 13- to 17-weeks old were used in the experiments, and HO was induced by a pinch injury as previously described [21]. From 7 days before the pinch injury, mice were fed water supplemented with 2 mg/mL doxycycline and 10 mg/mL sucrose, and the left gastrocnemius muscle was pinched using tissue forceps for 5 s. Tissue samples were collected 14 days after the pinch injury from mice euthanized using carbon dioxide (CO_2_). The age and body weight at the start point of each experiment were matched between groups.

### Induction of HO in wild-type mice

HO was induced in wild-type (WT) mice by collagenase injection into the Achilles tendon as previously described [22]. Eight-week-old male C57BL/6NJcl mice (Clea Japan) were used, and 20 μL of 1% collagenase (FUJIFILM Wako)/PBS was injected into their Achilles tendon under anesthesia using a mixture of medetomidine, midazolam, and butorphanol. Six weeks after the injection, tissue samples were collected after euthanization by CO_2_.

### Histological analysis

Collected tissue samples from mice were fixed with 4% paraformaldehyde for 48 h, embedded in paraffin, sectioned, and stained with hematoxylin and eosin (H&E) and Safranin O. Mice tissue sections were also processed as above with F4/80 (Abcam) and LYVE-1 antibody (Abcam) after deparaffinization using Clear Plus (Falma) without permeabilization. All samples were observed with a BZ-X810 (Keyence).

### Statistics

Statistical analysis was performed using JMP Pro 15 (SAS Institute Inc). Statistical significance was evaluated by two-way ANOVA followed by a Tukey–Kramer multiple comparison test or Dunnett's multiple comparison test. *P* values less than 0.05 were considered statistically significant. All studies were performed and analyzed with biological replicates.

## Results

### Establishment of FOP- and resFOP-ML

**Fig. 1:**
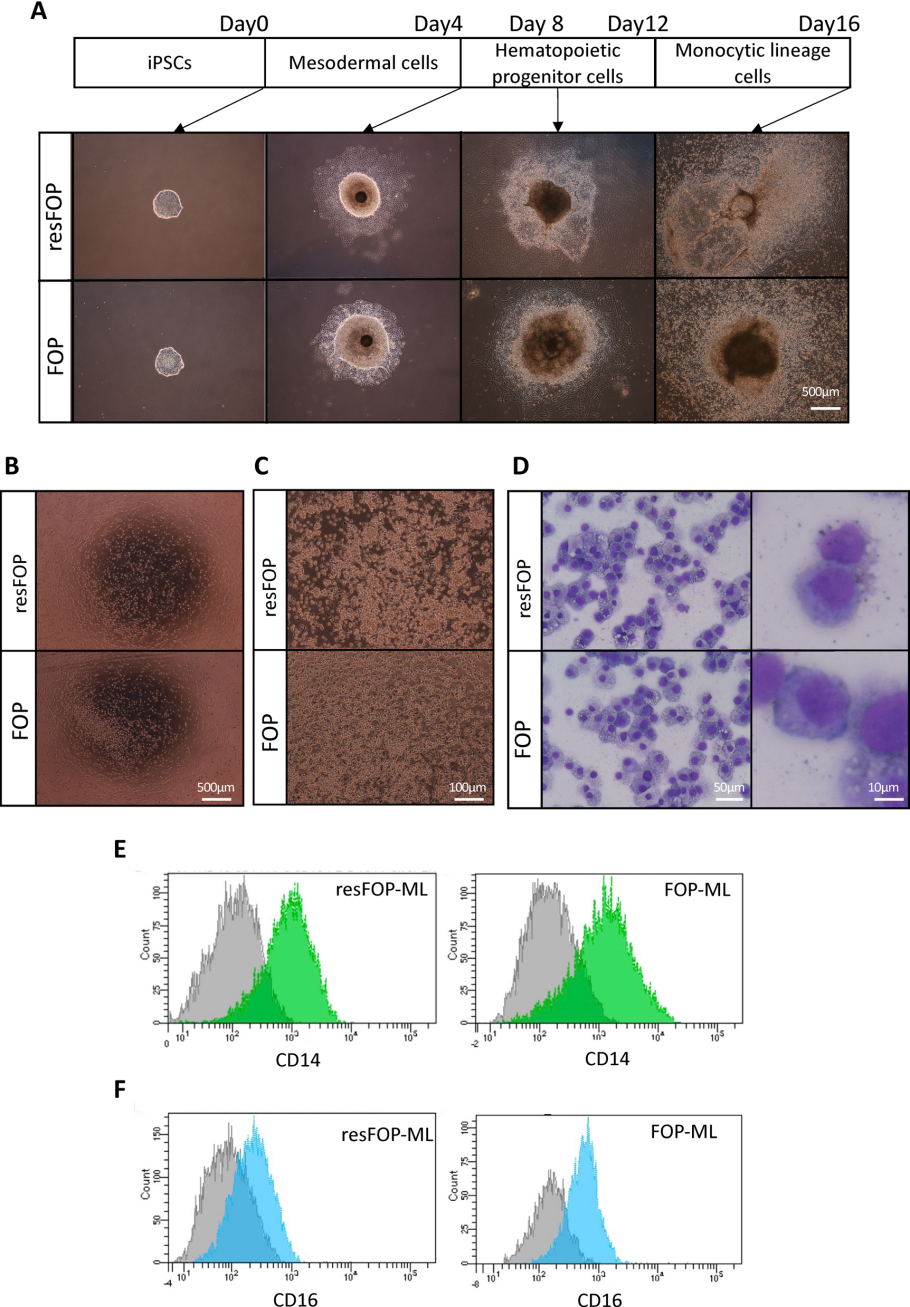
Establishment of immortalized monocytic lineage cell lines from FOP- and resFOP-iPSCs**. A**, Morphology of colonies during monocyte induction from iPSCs. Representative phase contrast images during the step-wise induction stages shown above. Scale bars = 500 μm. **B**, Representative phase contrast images of monocytes induced from each iPSC line. Scale bars = 500 μm. **C**, Representative phase contrast images of each ML. Scale bars = 100 μm. **D**, Representative morphology of each ML stained by May-Giemsa staining. Scale bars = 10 μm. **E**–**F**, Flow cytometric analyses of resFOP-ML and FOP-ML for the expression of CD14 (**E**) and CD16 (**F**)

Cellular and colony morphology during the monocyte induction showed no difference between FOP- and resFOP-iPSCs (Fig. 1A). After 18–21 days of monocyte induction, floating cells were collected, and CD14^+^ monocytes were sorted using MACS (Fig. 1B) and immortalized using lentivirus vectors encoding *BMI1*, *cMYC*, and *MDM2* genes [18, 19]. Proliferating CD14^+^ monocyte-derived cells were obtained from both FOP- and resFOP-iPSCs (FOP-ML and resFOP-ML) (Fig. 1C). The morphology of each cell line was compatible with those of primary monocytes, and there was no clear difference between them (Fig. 1D). The expression of CD14 in FOP-ML and resFOP-ML was further confirmed by FACS and showed an almost equal profile (Fig. 1E), whereas the population of cells expressing CD16 seemed to be larger in FOP-ML than in resFOP-ML (Fig. 1F).

### Characteristics of FOP-ML

**Fig. 2:**
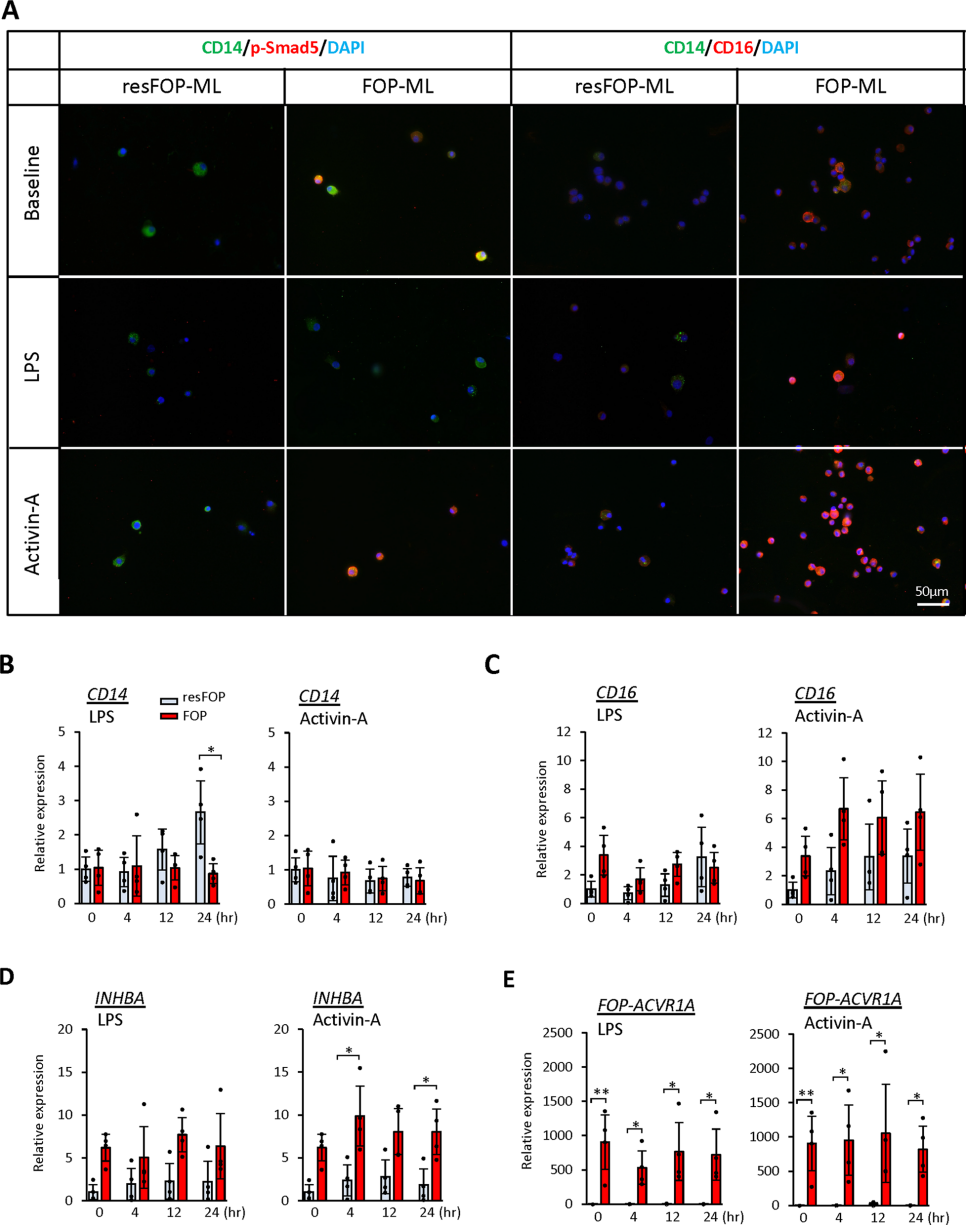
Characteristics of FOP- and resFOP-MLs with or without stimulations. FOP- and resFOP-MLs were treated with either LPS (10 ng/mL) or Activin-A (100 ng/mL) for 24 h. **A**, Immunostaining of CD14, CD16, and p-Smad5. Cells were stained before or after 24 h treatment of each chemical. Scale bar = 50 μm. **B**–**E**, Time course analyses of mRNA expressions aftere stimulation with LPS or Activin-A. RNAs were extracted at each time point and assessed for the expression of *CD14* (**B**), *CD16* (**C**), *INHBA* (**D**), and *FOP-ACVR1A* (**E**) by qPCR. The expression levels was shown as a value relative to those of resFOP-ML before treatment. The results were obtained from four biologically independent experiments. The error bars indicate standard deviation. Tukey–Kramer test *p < 0.05, **p < 0.01

To evaluate the effect of mutant ACVR1A on monocytes, we compared the characteristics of FOP- and resFOP-ML at baseline and under the stimulation of LPS or Activin-A. The phosphorylation of Smad5, a downstream marker of BMP signaling, was upregulated in FOP-ML at baseline and enhanced by Activin-A, but not in resFOP-ML (Fig. 2A). This result indicates that mutant ACVR1A-specific signaling was transduced in FOP-ML. CD16^+^ monocytes are regarded as a pro-inflammatory subpopulation [15]. Immunocytochemical staining showed that the proportion of CD16^+^ cells was higher in FOP-ML than resFOP-ML at baseline (Fig. 2A). LPS stimulation failed to increase the proportion of CD16^+^ cells in either group, but Activin-A stimulation induced CD16^+^ cells in both groups, although the induction was greater in FOP-ML (Fig. 2A). A qPCR analysis of *CD14* and *CD16* genes showed compatible results with those of the immunostaining. The baseline expression of *CD14* showed no difference between resFOP-ML and FOP-ML, and LPS increased the expression of *CD14* in resFOP-ML but not in FOP-ML, whereas Activin-A induced no changes in either cell line (Fig. 2B). In the case of *CD16*, however, its baseline expression in FOP-ML was higher than in resFOP-ML, and Activin-A increased the expression in both cell lines over time (Fig. 2C). These results suggested that FOP-ML may receive an Activin-A signal at baseline. In agreement with this hypothesis, the expression of *INHBA* gene, which encodes the alpha subunit of Activin-A, was much higher in FOP-ML than in resFOP-ML at baseline and continued to be highly expressed during treatment with Activin-A (Fig. 2D). The expression of *FOP-ACVR1A* gene showed no change during the culture period (Fig. 2E).

### Gene expression profiles of resFOP-ML and FOP-ML before and after stimulation

**Fig. 3:**
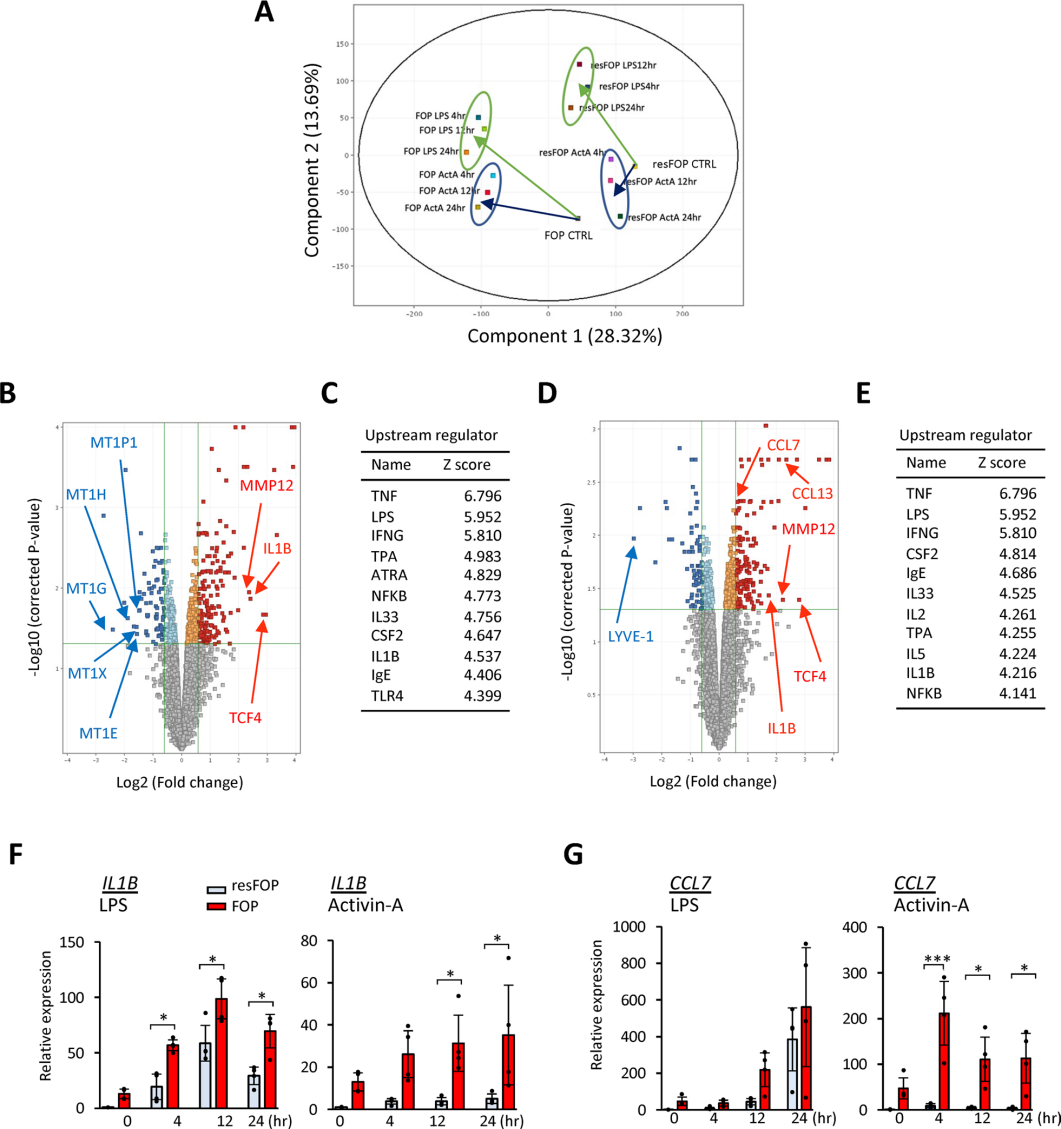
Gene expression profiles of FOP- and resFOP-ML before and after stimulation. **A**, Principle component analysis. FOP- and resFOP-MLs were treated with LPS (10 ng/mL) or Activin-A (100 ng/mL) for 24 h and RNAs were extracted at each time point from three biologically independent experiments and processed for microarray analysis. Green and blue circles enclose samples treated with LPS and Activin-A, respectively. Green and blue arrows indicate the migration from the control sample (without treatment). **B**–**E**, Volcano plots and the lists of upstream regulators. The expression level of each gene was compared between FOP-ML and resFOP-ML at baseline (**B**) and after stimulation with Activin-A for 12 h (**D**). Representative up-regulated (red) and down-regulated (blue) genes are shown (cutoff: fold change greater than 1.2; p value less than 0.05). The list of upstream regulators identified by IPA at each comparison are shown with Z-score (**C** and **E**). **F**–**G**, Time course analysis of mRNA expression after stimulation with LPS or Activin-A. RNAs were extracted at each time point and assessed for the expression of *IIL1B* (**F**), and *CCL7*(**G**) genes by quantitative reverse transcriptase PCR (qPCR). The expression levels were normalized to those of resFOP-ML before treatment. The results were obtained in four biologically independent experiments. Tukey–Kramer test *p < 0.05, ***p < 0.001

To investigate the effect of mutant ACVR1A on FOP-ML in detail, the entire gene expression profile was compared between FOP- and resFOP-ML by microarray. PCA demonstrated a clear difference between the two groups at baseline (Fig. 3A). After stimulation with LPS, a significant shift (Fig. 3A, indicated by the green arrows) was observed in both FOP- and resFOP-ML, showing movement with a similar direction and distance in the PC1 or PC2 component. The shift after Activin-A stimulation, however, showed a significant difference between the two (Fig. 3A, indicated by the black arrows). resFOP-ML showed only little shift after the stimulation and moved toward FOP-ML at baseline. On the other hand, FOP-ML showed a significant shift in the PC1 component and approached the position after LPS stimulation.

The transition of the gene profiles during the 24-h treatment was compared by clustering using the expression profile of genes up-regulated by LPS in both resFOP- and FOP-ML, such as *IL1B* and *IL6* (Additional file 2: Fig. S1). As for LPS-treated samples, both FOP- and resFOP-ML were found in the same cluster, and a heatmap showed a similar intensity of representative genes. On the other hand, FOP-ML and resFOP-ML created cell-type-specific clusters after treatment with Activin-A, and the intensity of cluster-defined genes was significantly different. These results suggested the FOP-ML-specific features are Activin-A dependent.

### Identification of Activin-A-induced features in FOP-ML

Volcano plots visualized up- and down-regulated genes in FOP-ML when compared with resFOP-ML at baseline (Fig. 3B) or after Activin-A stimulation (Fig. 3D). *IL1B*, *TCF4*, and *MMP12* genes were found among the up-regulated genes at baseline. TCF4 is a transcription factor that transduces the Wnt/β-catenin signal and is reported to be expressed in CD16^+^ pro-inflammatory monocytes [23]. MMP12, a member of the matrix protease family, is secreted by pro-inflammatory macrophages [24] and regulated by NFκB and β-catenin [25]. IL1β is a pro-inflammatory cytokine secreted by activated monocytes and macrophages and plays a key role in inflammatory responses [26]. One of the signals regulating the expression of *IL1B* is the non-canonical BMP signal in association with PU.1 [27], which has been shown to be expressed in pro-inflammatory monocytes [28]. A number of metallothionein genes were found among the down-regulated genes; these genes have been shown to inhibit the differentiation of monocytes [29] and are negatively regulated by the TGFβ signal via PU.1 [30, 31]. IPA identified several signal pathways that promote monocyte activity as upstream pathways in FOP-ML (Fig. 3C) [28], indicating that FOP-ML at baseline is already activated.

A volcano plot after 12 h of Activin-A stimulation demonstrated newly up-regulated genes, such as *CCL7* and *CCL13* (Fig. 3D). CCL7, also known as monocyte chemoattractant protein 3, is a secreted chemokine which directs chemotaxis in monocytes during inflammation [32]. CCL13, also known as monocyte chemoattractant protein 4, is also a monocytic chemokine with chemotactic activity [33]. The IPA-listed upstream regulators showed almost the same signals identified at baseline (Fig. 3E), further confirming that FOP-ML received the Activin-A signal at baseline.

The role of Activin A for the up-regulation of these genes was further investigated by comparing resFOP- and FOP-ML (Fig. 3F and G). LPS induced the expression of these genes in both cell lines in a time-dependent manner, but Activin-A induced these genes earlier and more in FOP-ML than in resFOP-ML, suggesting signals via mutant ACVR1A are involved in the up-regulation of these genes.

### TGFβ and BMP signals for the regulation of genes in FOP-ML

**Fig. 4:**
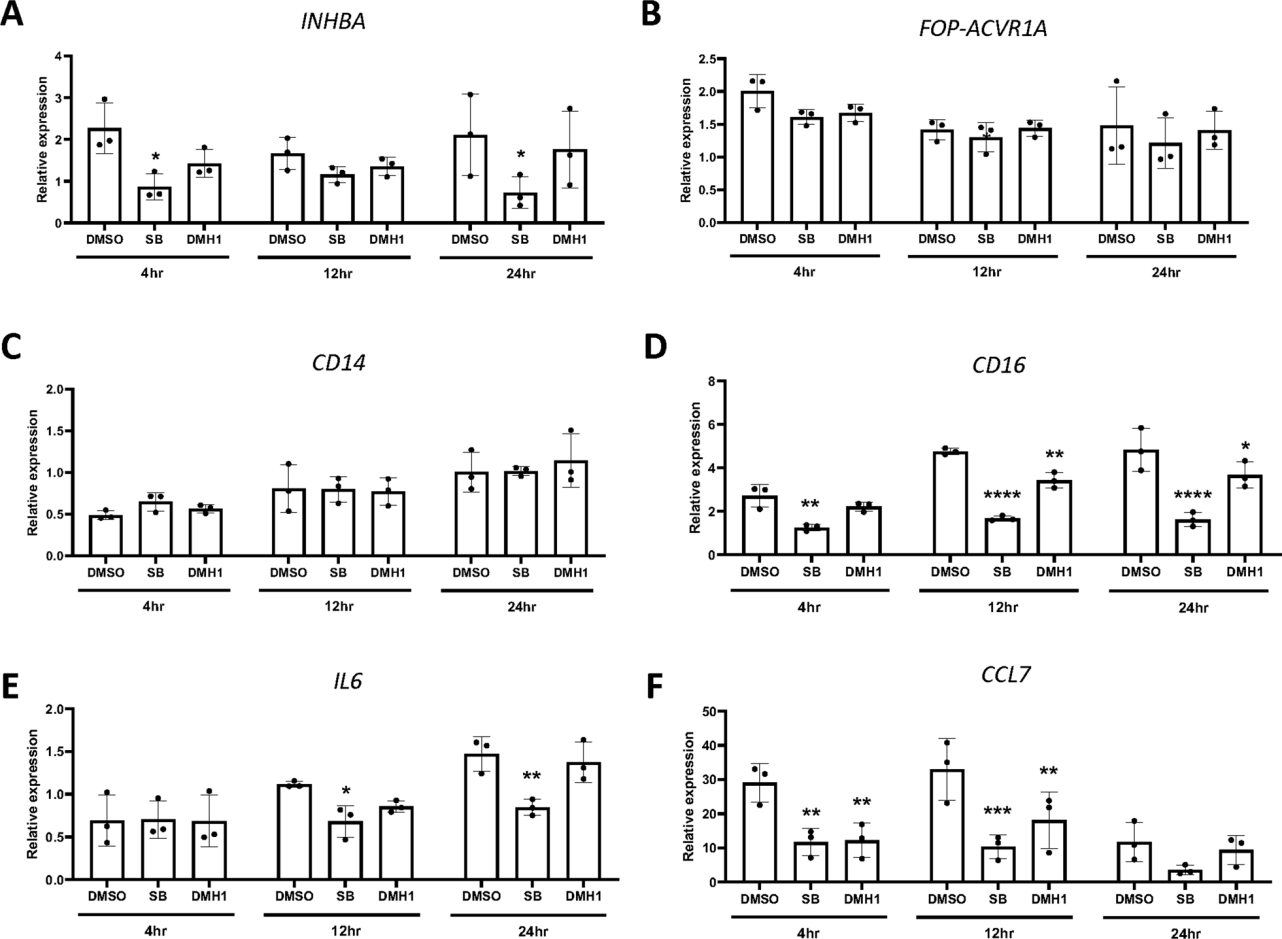
Effect of TGFβ or BMP signal inhibitors on the expression of Activin-A-induced genes in FOP-ML**.** FOP-MLs were treated simultaneously with Activin-A (100 ng/mL) and SB431542 (SB) (5 µM) or DMH1 (5 µM). RNAs were extracted at each time point and assessed for the expression of *INHBA* (**A**), *FOP-ACVR1A* (**B**), *CD14* (**C**), *CD16* (**D**), *IL6* (**E**), and *CCL7* (**F**) genes by quantitative reverse transcriptase PCR (qPCR). The expression levels were normalized to those of FOP-ML before treatment. The samples were collected from three biologically independent experiments. Two-way ANOVA followed by Dunnett's multiple comparison test (vs DMSO group). *p < 0.05, **p < 0.01, ***p < 0.001, ****p < 0.0001

Activin-A transduces both TGF and BMP signals in FOP cells [7]. To investigate the signal responsible for the feature of FOP-ML, Activin-A-treated FOP-ML were treated with an inhibitor for the TGFβ (SB) or BMP (DMH1) signal. The expression of *INHBA* was reduced by SB but not by DMH1, indicating that the induction of *INHBA* by Activin-A is mainly via the TGFβ signal pathway (Fig. 4A). Neither inhibitor changed the expression of *FOP-ACVR1* (Fig. 4B). The expression of *CD14* showed no difference by either inhibitor treatment (Fig. 4C). On the other hand, the expression of *CD16* gene induced by Activin-A was inhibited by SB at an earlier time point than by DMH1 (Fig. 4D). SB also inhibited the gradually increased expression of *IL6* by Activin-A (Fig. 4E). In contrast, the expression of *CCL7* gene was suppressed by both inhibitors even at an earlier time, suggesting the direct involvement of both signals for the regulation of this gene (Fig. 4F).

### Effect of corticosteroid on activated FOP-ML

**Fig. 5:**
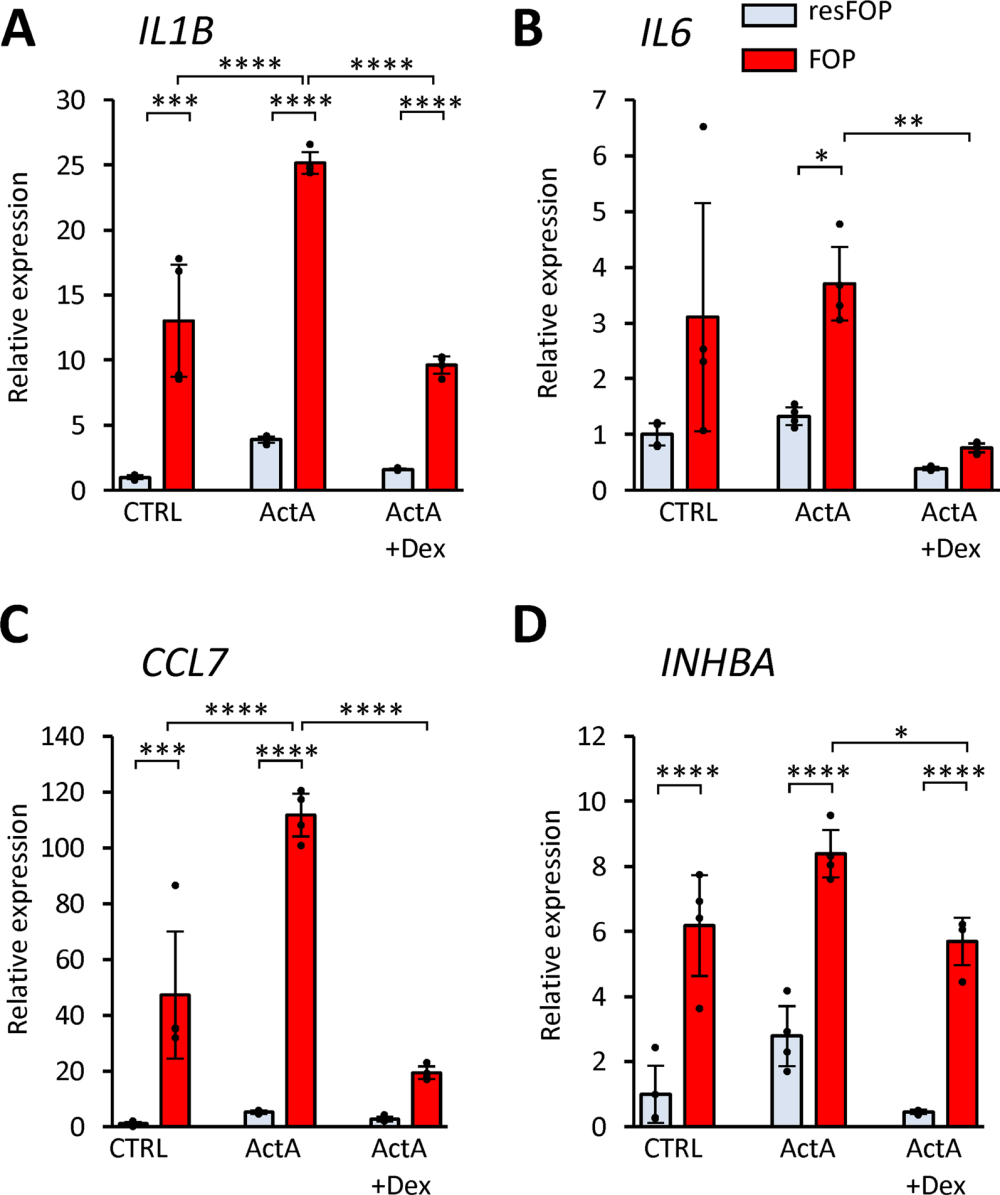
Effect of corticosteroid on gene expression induced by Activin-A. Cells were treated with Activin-A (100 ng/mL) and dexamethasone (1 µM) for 12 h and the expressions of *IL1B* (**A**), *IL6* (**B**), *CCL7* (**C**), and *INHBA* (**D**) were analysed by qPCR. The expression levels were normalized to those of resFOP-ML before treatment. The data were obtained from four biologically independent experiments. Tukey–Kramer test *p < 0.05, **p < 0.01, ***p < 0.001, ****p < 0.0001

To investigate whether the induced expression of these genes by Activin-A can be controlled by drugs, Activin-A-treated cells were simultaneously treated with dexamethasone, which is one of several corticosteroids currently used as Class I mediations for FOP patients, especially at the flare-up [34]. The up-regulation of *IL1B*, *IL6* and *CCL7* genes by Activin-A in FOP-ML was inhibited by dexamethasone (Figs. 5A–C), but the expression of *INHBA* was negligibly affected (Fig. 5D), suggesting the limited therapeutic effects of corticosteroids for FOP.

### Identification of target genes regulated by Activin-A in FOP-ML

**Fig. 6:**
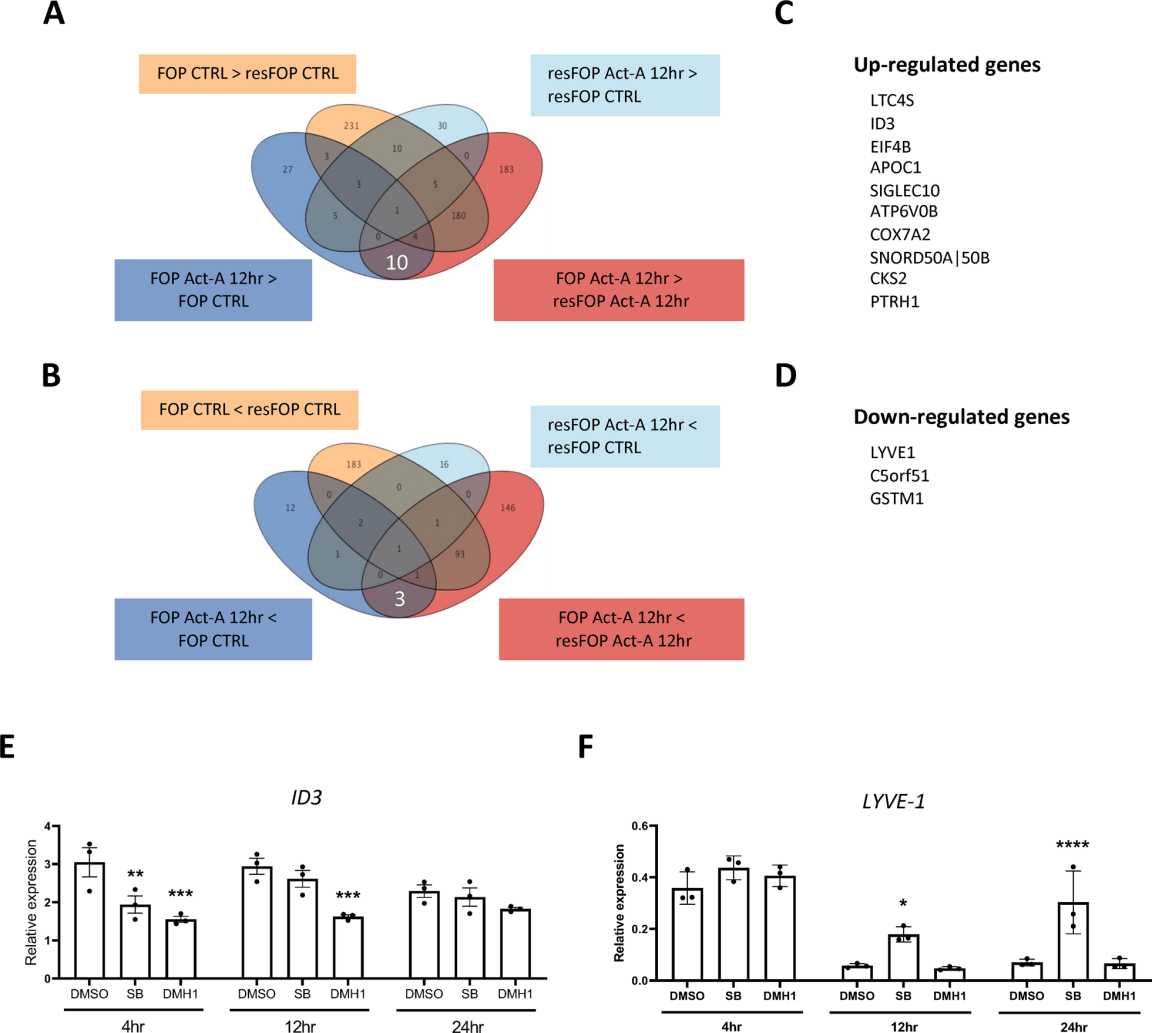
Identification of target genes regulated by Activin-A in FOP-ML. **A** and **B**, Venn diagrams showing the overlap of DEGs between different comparison groups for up-regulated (**A**) or down-regulated (**B**) genes. **C** and **D**, The list of up- (**C**) and down-regulated (**D**) genes. **E** and **F**, Effect of TGFβ and BMP signal inhibitors on the expression of *ID3* (**E**) or *LYVE-1* (**F**) genes in Activin-A treated FOP-ML. Cells were treated simultaneously with Activin-A (100 ng/mL) and SB431542 (SB) (5 µM) or DMH1 (5 µM). RNAs were extracted at each time point and assessed by quantitative reverse transcriptase PCR (qPCR). The expression levels were normalized to those of FOP-ML before treatment. The samples were collected from three biologically independent experiments. Two-way ANOVA followed by Dunnett's multiple comparison test (vs. DMSO group). *p < 0.05, **p < 0.01, ***p < 0.001, ****p < 0.0001

Using Venn diagrams, genes regulated by Activin-A in FOP-ML were searched (Fig. 6A and B), and 10 up-regulated (Fig. 6C) and 3 down-regulated genes (Fig. 6D) were identified. *EIF4B*, *ID3*, and *LTC4S* were among the up-regulated genes. EIF4B (eukaryote initiation factor 4B) is a member of the EIF family, which regulates translation in general and is one of the downstream molecules of the mTOR pathway [35]. The Ras-MAPK pathway was also shown to regulate its expression [36]. Since our previous study showed that FOP-ACVR1A abnormally transduces BMP signaling via the mTOR pathway in response to Activin-A [10], this result demonstrated that the FOP-ACVR1A-specific signal is transduced in FOP-ML. LTC4S is an enzyme that converts leukotriene A4 to create leukotriene C4, which is a mediator of anaphylaxis and inflammatory conditions [37], an important molecule in mast cells [38], and regulated by the ERK/NFκB pathway [39]. ID3 is a transcription factor and target gene of BMP, but Activin-A enhanced its expression, and DMH1 and SB suppressed it at earlier times (Fig. 6E).

One of the down-regulated genes, *LYVE-1*, encodes a receptor of hyaluronan [40]. Inhibition experiments indicated that the TGFβ signal is responsible for the suppression of this gene (Fig. 6F).

### Expression of LYVE-1 was down-regulated in monocytes with FOP-ACVR1 in vitro and in vivo

**Fig. 7:**
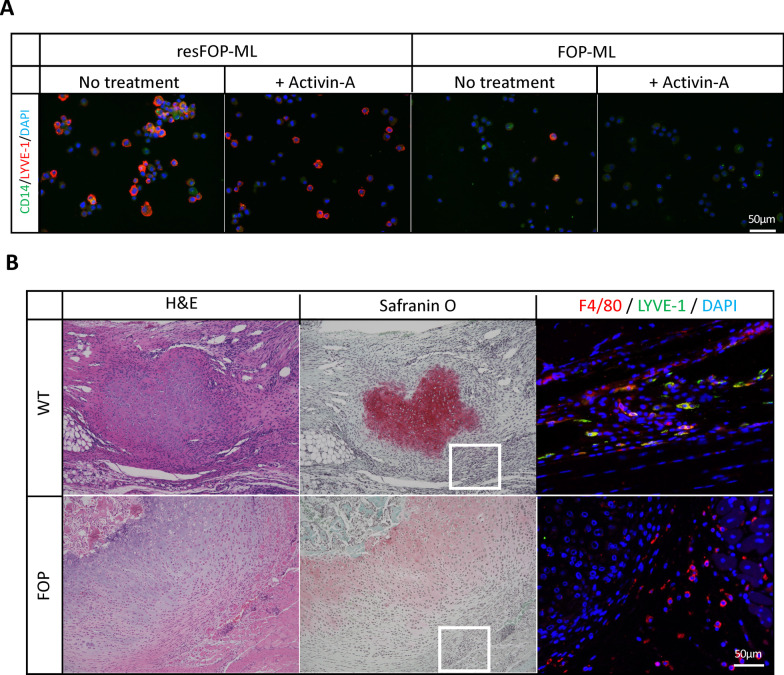
Identification of LYVE-1 as a down-regulated gene in vitro and in vivo. **A**, Expression of LYVE-1 in resFOP-ML and FOP-ML. Cells were treated with or without Activin-A for 12 h, and stained by anti-CD14, anti-LYVE-1 antibodies and DAPI. Scale bar = 50 μm. **B**, Expression of LYVE-1 in monocytes localized in HO lesions. Tissues were taken from collagenase injected sites (WT) or pinch-injured sites (FOP) and stained with H&E or Safranin-O. The expression of LYVE-1 in monocytes/macrophages localized adjacent to the HO site (white rectangles) was analyzed by anti-LYVE-1 antibody along with anti-F4/80 antibody and DAPI. The samples were collected from three biologically independent experiments. Scale bar = 250 μm

Although LYVE-1 was originally expected to be expressed selectively in lymphangitic cells [40], recent data demonstrated its expression in monocytes/macrophages and its involvement in matrix formation [41]. An immunocytochemical analysis showed the expression of LYVE-1 in resFOP-ML, but hardly in FOP-ML after treatment with Activin-A (Fig. 7A). Because the putative function of LYVE-1 is related to matrix formation [42], we further analyzed this molecule in vivo using pinch-injury-induced HO tissues from FOP-ACVR1A mice and collagenase-induced HO from WT mice (Fig. 7B). H&E and Safranin-O staining showed heterotopic cartilage formation in the Achilles tendon of WT mice, and F4/80 positive monocytes and macrophages were found adjacent to the HO, which was also positive for LYVE-1. On the contrary, F4/80 positive cells adjacent to heterotopic cartilage tissue developed at the injured site of FOP-ACVR1A mice were negative for LYVE-1. These in vivo results agree with the in vitro data, suggesting the usefulness of FOP-ML to identify the pathologic change in monocytes with FOP-ACVR1 and that FOP-ML are a promising tool to find new therapeutic approaches.

## Discussion

iPSCs derived from patients with a particular type of hereditary disease (disease-specific iPSCs) have been widely used to investigate the disease-causing mechanisms and develop therapeutic drugs [43]. There are several advantages to disease-specific iPSCs. The induction of target cells from the iPSCs can be repeated and therefore there is no limitation in the number of cells available for the analysis. Additionally, the effect of the genetic background for the phenotype can be avoided by making mutation-corrected iPSCs from each iPSC line as an isogenic control. Finally, multiple types of cells from the same iPSC line, in other words from the same patient, can be analyzed if the appropriate induction methods for each type of cells are available. We have been applying this strategy to a number of musculoskeletal diseases and successfully recapitulated the diseases in vitro and identified candidate drugs [44–47]. In the case of FOP, we induced MSCs from FOP- and resFOP-iPSCs, investigated the transition from MSCs to chondrocytes and identified Activin-A as a key factor to initiate the process of HO [7]. In the present report, we focused on the initial inflammation phase of HO and analyzed the effect of the ACVR1A mutation on monocytes by comparing the gene expressions of FOP-ML and resFOP-ML. We made several observations indicating that mutant ACVR1 contributs to the exaggerated of inflammation and possibly the matrix formation (Additional file 3: Fig. S2). FOP-ML showed a gene expression profile consistent with the pro-inflammatory status, as if they had been stimulated by inflammatory cytokines such as TNFα or LPS at baseline. These data agree with those of primary cells collected from FOP patients [13, 15]. In this regard, the up-regulation of *INHBA* gene may play a central role in the accelerated inflammatory status of FOP-ML at baseline and after stimulation. Several inflammatory-related genes, including *IL1B*, *MMP12*, and *TCF4*, were up-regulated at baseline in FOP-ML, while Activin-A stimulation induced the expression of *CCL7* and *CCL13* genes. Experiments using inhibitors indicated some of these genes are directly regulated by TGFβ and/or BMP signals induced by Activin-A, among which the expression of *CCL7* seemed to be regulated by both signals. Previously, we demonstrated that Activin-A induced both TGFβ and BMP signals in FOP-MSCs [10]. *CCL7* may be one of molecules regulated by both signaling pathways in monocytes with FOP-ACVR1A. In contrast, the regulation of other genes is not simple. LPS is known to induce *IL1B* gene via the NFkB signal [48]. We found Activin-A induced the expression of *IL1B* in FOP-ML but not in resFOP-ML, suggesting the dual signals induced by Activin-A in FOP-ML may crosstalk with the NFkB signal pathway [49].

FOP-ML are also a useful tool for the search of therapeutic targets in the initial inflammatory stage of FOP. We identified LTC4S as an Activin-A-regulated molecule through microarray analysis, suggesting an increase of leukotriene production in FOP-ML. Although the therapeutic effect of leukotriene inhibitors is limited and the drugs are categorized as class II medication for flare-up [34], our data suggest the prophylactic use of leukotriene inhibitors for suppressing the early event of HO. Dexamethasone, class I medication, suppressed the expression of inflammatory cytokines in FOP-ML, however it did not have an inhibitory effect on the abnormal INHBA expression in FOP-ML. Higher Activin-A production from FOP patient derived M1 macrophages was also reported in a previous report [50]. A treatment that can inhibit the higher expression of INHBA in FOP monocytes would be prospectively ameliorate HO formation and could be found from our FOP-ML via high-throughput screening. The identification of LYVE-1 as a gene down-regulated by Activin-A is an intriguing finding when its function in matrix formation is considered. LYVE-1 is a marker for distinguishing between blood and lymphatic vessels and plays an important role in leukocyte trafficking [40]. Recent data demonstrated that LVYE-1 is also expressed on monocytes/macrophages, which exist not only around the arteries but also in skeletal muscle [41]. LYVE-1 on macrophages activates MMP-9 by engaging hyaluronic acid and maintains the elasticity of the arterial wall by the MMP-9-dependent degradation of collagen [20]. In the present report, for the first time, we demonstrated that monocytes/macrophages localizing adjacent to HO tissues express LYVE-1 in the collagenase-induced HO model. Although the significance of this expression is not yet clear, the proposed function of LYVE-1 for the degradation of collagens may contribute to the limited HO formation in this model. On the other hand, almost no expression of LYVE-1 was found in monocytes/macrophages localizing adjacent to HO tissues in FOP mice. Considering the suppression of LYVE-1 expression by Activin-A and the elevated expression of *INHBA* gene in FOP-ML, monocytes/macrophages in FOP mice may contribute to uncontrolled HO formation by the loss of LYVE-1 expression, which results in the failure of collagen degradation (Additional file 3: Fig. S2). Although further experiments are necessary, these data suggest LYVE-1 as a new target for FOP therapy.

## Conclusion

In this study, we established immortalized monocyte cell lines from FOP- and resFOP-iPSCs (FOP-ML and resFOP-ML, respectively) and demonstrated the pro-inflammatory status of the former. Most features of FOP-ML are compatible with those observed in primary monocytes collected from FOP patients, validating the use of FOP-ML as an unlimited cellular source for FOP study.

## Supplementary Information

**Additional file 1**:** Table S1**. Primers for qRT-PCR.

**Additional file 2**:** Fig S1**. Hierarchical clustering of samples treated with LPS or Activin-A.

**Additional file 3**:** Fig S2**. Schematic summary of the inflammatory signals in FOP-ML.

## Data Availability

The data used during this study are available from the corresponding author on reasonable request.

## Abbreviations

ACVR1A: Activin A receptor type 1A
ACVR1B: Activin A receptor type 1B
CCL7: C–C motif chemokine ligand 7
FOP: Fibrodysplasia ossificans progressiva
H&E: Hematoxylin and eosin
HO: Heterotopic ossification
IL1β: Interleukin 1 beta
INHBA: Inhibin subunit beta A
iPSCs: Induced pluripotent stem cells
ML: Monocyte cell line
MSC: Mesenchymal stromal cell
LPS: Lipopolysaccharide
