# Supplementary material for: Recapitulation of pro-inflammatory signature of monocytes with ACVR1A mutation using FOP patient-derived iPSCs
Source: Orphanet J Rare Dis. 2022 Sep 21;17:364. doi: 10.1186/s13023-022-02506-3 (PMC9494870; doi:10.1186/s13023-022-02506-3)
Supplement: Supplementary file 1 — Additional file 1: Table S1. Primers for qRT-PCR. [file 13023_2022_2506_MOESM1_ESM.pdf]

Table S1. Primers for qRT-PCR

GENE	GENE FULL NAME	PRIMER SEQUENCE 5'-3'	
ACTB	*Actin Beta*	Forward	CACCATTGGCAATGAGCGGTTC
		Reverse	AGGTCTTTGCGGATGTCCACGT
ACVR1	*Activin receptor-like kinase-2*	Forward	TGGTACAAAGAACAGTGGCTAG
		Reverse	CCATACCTGCCTTTCCCGA
FOP-ACVR1	*Activin receptor-like kinase-2 (R206H)*	Forward	TGGTACAAAGAACAGTGGCTTA
		Reverse	CCATACCTGCCTTTCCCGA
CD14	*Cluster of differentiation 14*	Forward	ACTTGCACTTTCCAGCTTGCG
		Reverse	CCTGTGGGCGTCTCCATTCC
CD16	*Cluster of differentiation 16*	Forward	ATGTGTCTTCAGAGACTGTGAAC
		Reverse	TTTATGGTCCTTCCAGTCTCTTG
IL1B	*Interleukin 1B*	Forward	CAGCTACGAATCTCCGACCAC
		Reverse	GGCAGGGAACCAGCATCTTC
IL6	*Interleukin 6*	Forward	AACCTGAACCTTCCAAAGATGG
		Reverse	TCTGGCTTGTTCCTCACTACT
INHBA	*Inhibin subunit beta A*	Forward	GAGGTTGGCAAAGGGGCTAT
		Reverse	TGGATCATTGCTCCCTCTGG

ID1	*Inhibitor of DNA binding 1*	Forward	CCAACGCGCCTCGCCGGATC
		Reverse	CTCCTCGCCAGTGCCTCAG
LYVE1	*Lymphatic vessel endothelial hyaluronan receptor 1*	Forward	AGGCTAGGTGGGTTGAAAGC
		Reverse	CTTACCGGGCTCAGAAGGAC
LCT4S	*Leukotriene C4 synthase*	Forward	ACGAGGTAGCTCTACTGGCT
		Reverse	ACGAGGTAGCTCTACTGGCT
ID3	*Inhibitor of DNA binding 3*	Forward	GGAGCTTTTGCCACTGACTC
		Reverse	TTCAGGCCACAAGTTCACAG
EIF4B	*Eukaryotic translation initiation factor 4B*	Forward	TTCCCAAATCGCCACCCTAC
		Reverse	TGCTGGGTTCACGTGGTAAA
CCL7	*CC chemokine ligand 7*	Forward	TTGCTCAGCCAGTTGGGATTA
		Reverse	AGTGGCTACTGGTGGTCCTT
