# Supplementary figures and images for: Recapitulation of pro-inflammatory signature of monocytes with ACVR1A mutation using FOP patient-derived iPSCs
Source: Orphanet J Rare Dis. 2022 Sep 21;17:364. doi: 10.1186/s13023-022-02506-3 (PMC9494870; doi:10.1186/s13023-022-02506-3)

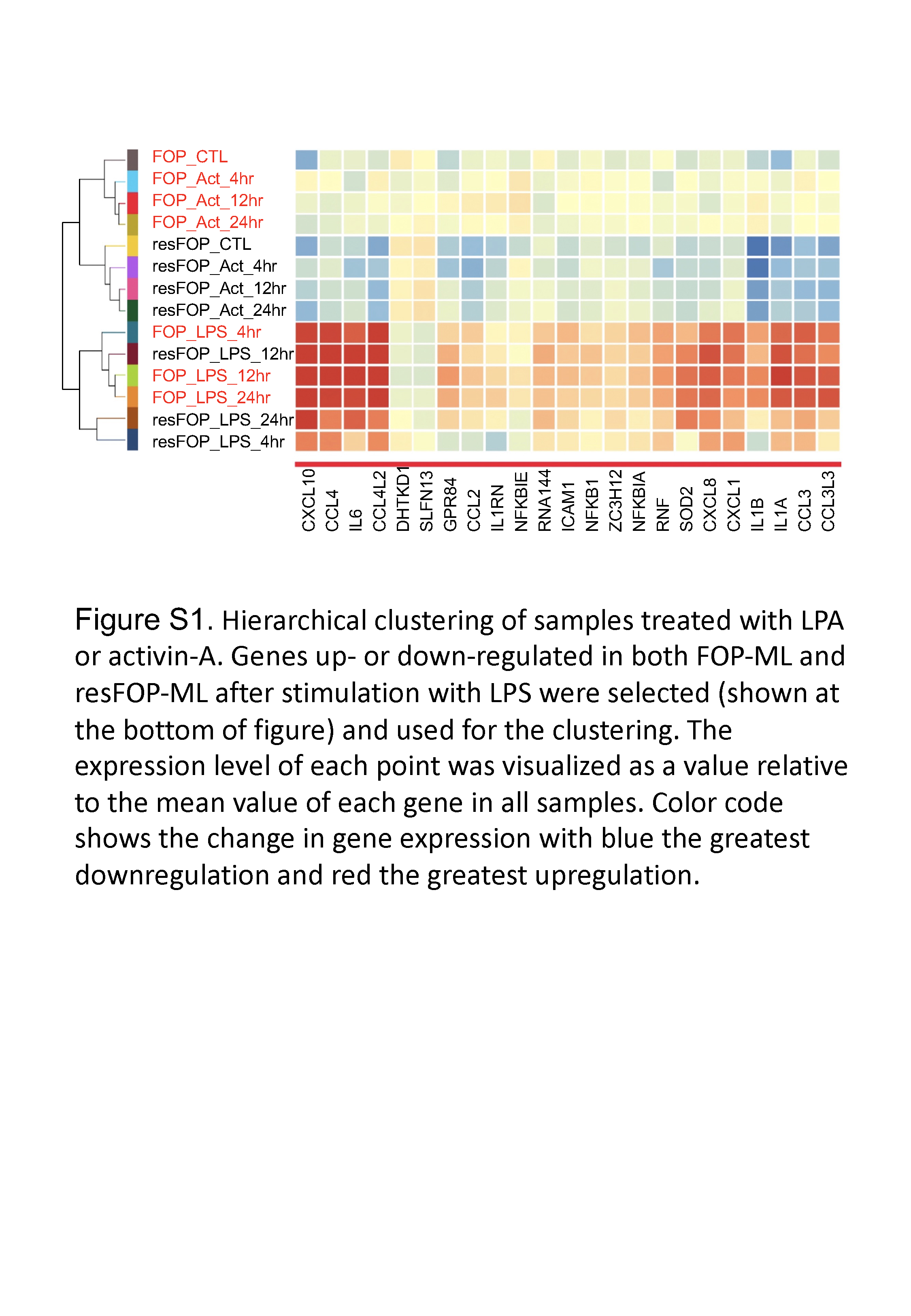

Supplement: Supplementary file 2 — Additional file 2: Fig S1. Hierarchical clustering of samples treated with LPS or Activin-A. [file 13023_2022_2506_MOESM2_ESM.tiff]

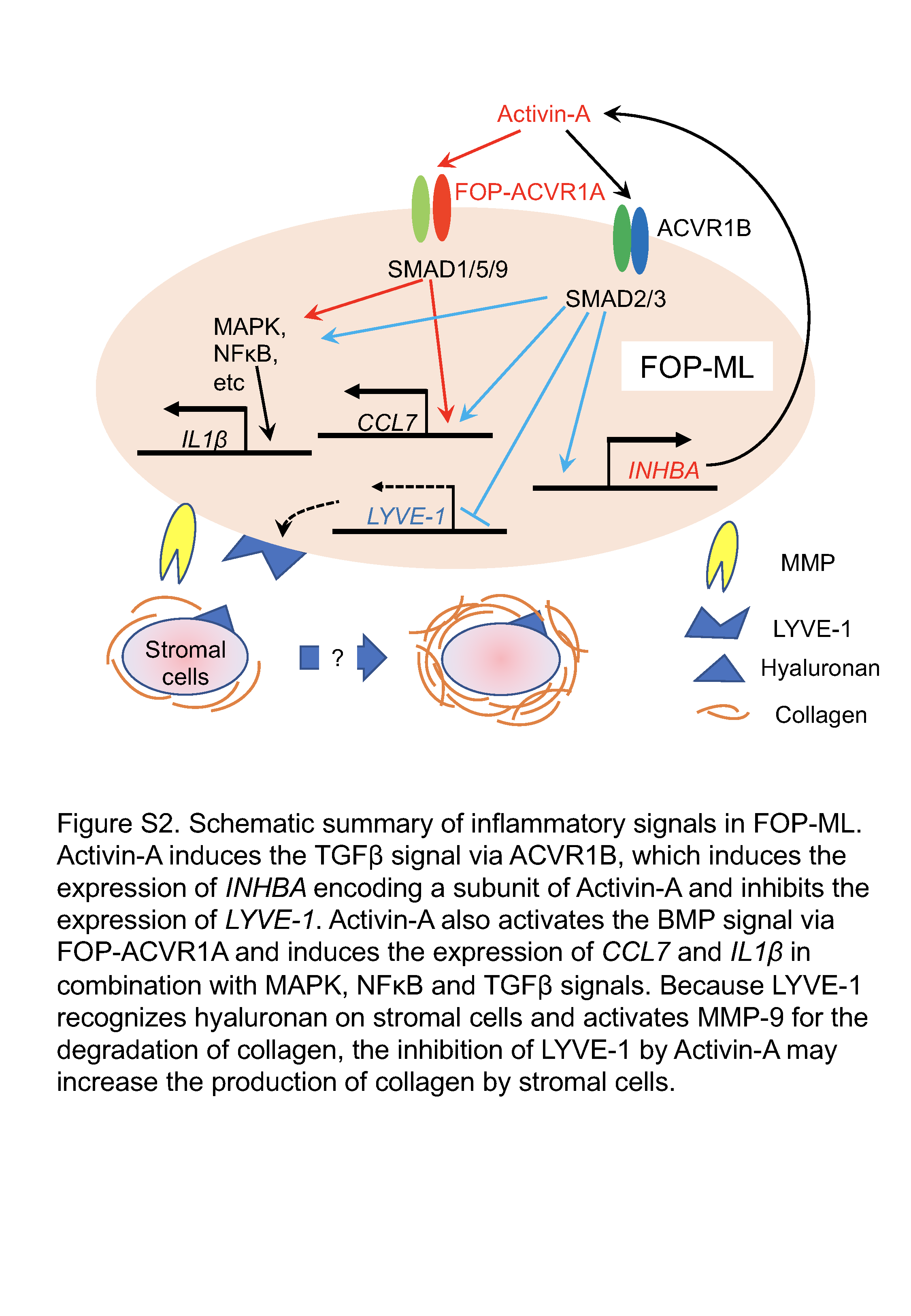

Supplement: Supplementary file 3 — Additional file 3: Fig S2. Schematic summary of the inflammatory signals in FOP-ML. [file 13023_2022_2506_MOESM3_ESM.tiff]
